# Flavonoid ingredients of *Ginkgo biloba* leaf extract regulate lipid metabolism through Sp1-mediated carnitine palmitoyltranferase 1A up-regulation

**DOI:** 10.1186/s12929-014-0087-x

**Published:** 2014-09-03

**Authors:** Ting Wei, Fei-fei Xiong, Shi-dong Wang, Ke Wang, Yong-yu Zhang, Qing-hua Zhang

**Affiliations:** Research Center for Traditional Chinese Medicine and Systems Biology, Shanghai University of Traditional Chinese Medicine, Shanghai, China; School of Life Science and Technology, Tongji University, Shanghai, China; Shanghai-MOST Key Laboratory of Health and Disease Genomics, National Engineering Center for Biochip at Shanghai, Shanghai, China; State Key Laboratory of Medical Genomics and Shanghai Institute of Hematology, Ruijin Hospital, Shanghai Jiaotong University School of Medicine, Shanghai, China

**Keywords:** *Ginkgo biloba* extract (GBE), CPT1A, Sp1, Flavonoid ingredients, Regulation

## Abstract

**Background:**

Lipid accumulation is the primary evidence of non-alcoholic fatty liver disease (NAFLD). *Ginkgo biloba* extract (GBE) and its flavonoid ingredients (quercetin, kaempferol, and isorhamnetin) could lessen the lipid accumulation associated with up-regulation of the rate-limiting enzyme, carnitine palmitoyltransferase 1A (CPT1A), in the β-oxidation of long-chain fatty acids. In this study, we investigated the mechanisms by which GBE and its flavonoids induced expression of CPT1A.

**Results:**

CPT1A inhibition with RNAi resulted in triglyceride accumulation in HepG2 cells. Through deletion and mutation analysis of CPT1A’s promoter combined with electrophoretic mobility shift assay (EMSA) and chromatin immunoprecipitation (ChIP) experiments, the CPT1A promoter region (−50 to −5 nt) was determined to contain two putative Sp1 binding sites, namely Sp1a and Sp1b, which might act as the GBE regulation response DNA element. Sp1 might be induced to transfer from cytoplasma to nucleus to bind the promoter region of −50 to −5 nt by GBE. The regulatory effects of GBE on CPT1A were also verified on the flavonoid ingredients quercetin, kaempferol, and isorhamnetin.

**Conclusion:**

Sp1 was crucial in regulating CPT1A expression with GBE and its flavonoid ingredients, and the −50 to −5 nt region of CPT1A promoter played important roles in Sp1 binding.

**Electronic supplementary material:**

The online version of this article (doi:10.1186/s12929-014-0087-x) contains supplementary material, which is available to authorized users.

## Background

Non-alcoholic fatty liver disease (NAFLD) is characterized by triglyceride (TG) accumulation in hepatocytes and is commonly associated with dyslipidemia, hypertension, obesity, and hyperglycemia. To date, there are no ideal NAFLD treatment options. Traditional strategies mainly focus on lifestyle modification and body weight loss, simply slowing steatosis with no long-term efficacy [1]. Although various drugs were under investigation, efficacy and safety profiles remained uncertain, and no proven treatments have yet been approved [2].

*Ginkgo biloba* has been used for hundreds of years in China to treat various disorders. EGb761, *Ginkgo biloba* leaf extract, is widely used as a dietary supplement or phytomedicine currently in western countries. *Ginkgo biloba* extract (GBE) mainly consists of two groups of active components: flavonoid and terpenoid [3]. Our previous work has shown that GBE regulates lipid metabolism and lessens the lipid accumulation in the livers of rats fed a high-fat diet (HFD) or hepatocytes at the transcriptome regulation level. GBE, with its flavonoid ingredients, could significantly up-regulate expression of carnitine palmitoyltransferase 1A (CPT1A), a rate-limiting enzyme in the β-oxidation of long-chain fatty acids (LCFAs), and elevate its activity [4,5]. However, the mechanisms of regulation in CPT1A expression remained uncertain.

CPT1 is located in the outer mitochondrial membrane and facilitates the transport of long-chain fatty acids into the mitochondria for β-oxidation by converting them from acyl-CoA into acyl-carnitine [6]. In the liver, CPT1A is the primary isoform expressed while CPT1B and CPT1C specifically distribute into muscle, heart, and brain [7,8]. Alteration of CPT1A occurs in response to lipid metabolites, hormones, nutrition, among others. Soy isoflavones and L-carnitine regulate CPT1A activity in HepG2 cells positively [9]. Peroxisome proliferator-activated receptor α (PPARα) rapidly increases CPT1A expression in rat [10]. Moderate increases in CPT1A activity causes profound effects on fatty acid oxidation and is sufficient to reduce hepatic triglyceride accumulation, both *in vivo* and *in vitro*, with an unexpected mitigating effect on lipid-induced insulin resistance [11–13].

In the present study, we focused on the regulation of CPT1A expression by GBE, and attempted to reveal its underlying mechanisms. Thus, RNAi technology was used to evaluate the role of CPT1A in GBE’s effect. In addition, nucleotide deletion and mutation analysis of a CPT1A promoter combined with electrophoretic mobility shift assay (EMSA) and chromatin immunoprecipitation (ChIP) experiments were conducted to identify the specific responsive element of GBE and its flavonoids. Our results finally revealed an Sp1 binding region located in the CPT1A promoter was critical for the regulation of CPT1A by GBE.

## Methods

### Cell culture and treatment

The human hepatocellular carcinoma cell line, HepG2, was kindly gifted by the Chinese National Human Genome Center at Shanghai. HepG2 cells were cultured in Minimum Essential Medium (MEM) (Invitrogen, Carlsbad, CA, USA) containing 10% fetal bovine serum (FBS) (Invitrogen) at 37°C in a 5% CO_2_ incubator. GBE was provided by Shanghai Xingling Science and Technology Pharmaceutical Co., Ltd. (Shanghai, China) as a standardized product that contained approximately 24% flavonol glycosides, 6% terpenelactones (ginkgolides, bilobalide), and less than 5 parts per million (ppm) of ginkgolic acid. Quercetin, kaempferol, and isorhamnetin were purchased from Shanghai Tauto Biotech Co., Ltd. (Shanghai, China).

For experiments, cells were seeded into a 24-well plate at a density of 1 × 10^5^ cells per well and incubated to reach about 70% confluence. Cells were then treated with MEM-2% FBS containing one of the following: 200 μg/ml GBE (G200), 20 μg/ml quercetin (Q20), 20 μg/ml kaempferol (K20), or 8 μg/ml isorhamnetin (I8) for 12 or 24 hours. The concentrations of the flavonoids were set according to their respective proportions in GBE. As control, 0.1% dimethyl sulfoxide (DMSO, D) was used.

### Cellular triglyceride content measurement

The cellular triglyceride content was measured as before [5]. After 24 hours of treatment, the harvested cells were washed with 1 × PBS, suspended in 500 μl isopropanol with sonication for 30 minutes and stood on ice for 1 hour. Then, samples were centrifuged at 13,200 rpm for 10 minutes at 4°C. The extracted supernatants were evaporated in a vacuum centrifuge concentrator and resuspended in 20 μl isopropanol for further cellular triglyceride content measurement using Total Triglyceride Detection Kit (Shanghai Kehua Bio-Engineering Co., Ltd., Shanghai, China). Meanwhile, the precipitates were lysed by RIPA lysis buffer (Beyotime, Haimen, Jiangsu, China), and total protein level was determined by BCA Protein Assay Kit (Beyotime). Triglyceride content (TG) was normalized to corresponding total protein.

### RNA extraction and real time RT-PCR

The total RNA was extracted from cells using TRIzol reagent (Invitrogen) according to the standard protocol. The purity and concentration of the extracted RNA samples were examined by a 2100 Bioanalyzer and the RNA 6000 LabChipR (Agilent Technologies, Santa Clara, CA, USA). Real-time RT-PCR was carried out on an ABI PRISM 7300 Sequence Detector (Applied Biosystems, Foster City, CA, USA) using SYBR green I fluorescent dye (Toyobo, Osaka, Japan). The primers used were as follows: For CPT1A (GenBank: NM_001876): forward, 5′-CGGTTGCTGATGACGGCTAT; reverse, 5′-CAAAGCGATGAGAATCCGTC, and for Sp1 (GenBank: NM_001251825.1): forward, 5′-CTGCCACCATGAGCGACCAAG; reverse, 5′- CTGATCTCAGAAGCCATTGC. For data analysis, a method designated as 2^-∆∆CT^ was used to calculate fold change. β-actin was used as an internal control. All samples were performed in triplicate, and the final data represented the mean of at least three individual experiments.

### Western blot

Whole-cell lysates prepared by RIPA lysis buffer (Beyotime) from HepG2 cells were separated by SDS-PAGE and then transferred onto PVDF membranes (Millipore, Billerica, MA, USA). The membranes were probed with anti-β-actin (1:2000; Proteintech, Chicago, IL, USA) or anti-CPT1A (1:250; Santa Cruz Biotechnology, Santa Cruz, CA, USA) and horseradish peroxidase (HRP) conjugated secondary antibody (1:2000; Santa Cruz Biotechnology) and then detected using ECL reagents (GE Healthcare, Piscataway, NJ, USA) by imaging systems. Nuclear and cytoplasmic proteins were extracted separately, while α-tubulin and histone H3 were used as internal controls.

### Luciferase reporter plasmid construction and site-directed mutagenesis

A series 5′-deletion fragments of the human CPT1A promoter with KpnI/HindIII restriction sites were PCR-amplified from human genomic DNA (Table 1), and constructed into KpnI/HindIII sites of pGL3-Basic vector (Promega, Madison, WI, USA) in front of the luciferase reporter gene. Three site-directed mutated constructs were also used to determine the responsive element of GBE and its flavonoids (Table 2). All constructs were validated with DNA sequencing.

**Table 1:** **Primers used in luciferase reporter plasmid construction**

**Primer name** ^**a**^	**Sequence(5′-to-3′)**
pCPT1A-1/Luc_F	GGGGTACCTTTCTTGTAGCTATGGTAGGC
pCPT1A-2/Luc_F	GGGGTACCGAAGAGCCCTGGGAACAGAC
pCPT1A-1 ~ 2/Luc_R	CCCAAGCTTCCCCTGATGGTATTCACCCCT
pCPT1A-3/Luc_F	GGGGTACCTTTCTTGTAGCTATGGTAGGC
pCPT1A-4/Luc_F	GGGGTACCTAAGGCCTCCCCAGTGCGG
pCPT1A-5/Luc_F	GGGGTACCGTCCCTGCCCCGCCCGGC
pCPT1A-6/Luc_F	GGGGTACCGGAAGGGCGCACGGT
pCPT1A-3 ~ 6/Luc_R	CCCAAGCTTGTCTGTTCCCAGGGCTCTTC

^a^_R: reverse primer, _F: forward primer.

**Table 2:** **Sequences of wild-type and mutant CPT1A promoter constructs in mutational analysis**

**Name**	**Sequence (46 bp)** ^**a**^		
	−50	Sp1a	Sp1b
Wild type	gtccctGCCCCGCCCGgcctgcaggtggcaccTAGGCGGCGCgcag		
Sp1a mut.	gtccctGCC**TAA**CCC**T**gcctgcaggtggcaccTAGGCGGCGCgcag		
Sp1b mut.	gtccctGCCCCGCCCGgcctgcaggtggcaccTAGG**TAA**CGCgcag		
Sp1a-b mut.	gtccctGCC**TAA**CCC**T**gcctgcaggtggcaccTAGG**TAA**CGCgcag		

^a^Putative cis-elements were indicated with capital, and introduced mutations were shown in bold.

### Transient transfection and luciferase assays

Lipofectamine™ 2000 (Invitrogen) was used for *in vitro* transfection of all the experimental vectors according to standard protocols. Briefly, 1 × 10^5^ HepG2 cells were seeded into 24-well culture plates and grown overnight to 80-90% confluence. For luciferase activity assays, 0.5 μg of reconstructed CPT1A-promoter reporter plasmids along with 10 ng of PRL-SV40 plasmids that encoded *renilla* luciferase for normalization were co-transfected into each well. Twenty-four hours after transfection, the culture media was changed with or without GBE for a continuous 24 hours. After treatment, cell lysates were collected and assayed for luciferase activity using a Dual-Luciferase Reporter Assay kit (Promega).

### RNA interference

Four shRNAs were designed to target the coding sequences of human CPT1A and cloned into pGPU6/GFP/Neo vectors (Table 3). Vectors without influence on the expression of CPT1A were also produced as negative control (NC). siRNA-Sp1 (5′-GCUCCAGAUCCAGUAUCUUTT-3′) was used to target Sp1. All shRNAs and siRNA-Sp1 were synthesized by Shanghai GenePharma Co., Ltd. (Shanghai, China).

**Table 3:** **Sense sequences of the oligonucleotides for synthesized shRNAs**

**shRNA name**	**Sense sequences**
shRNA1	5′-CACCGGATGGGTATGGTCAAGATCTTTCAAGACAAGATCTTGACCATACCCATCCTTTTTTG-3′
shRNA2	5′-CACCGCCTTTACGTGGTGTCTAAATTTCAAGAGAATTTAGACACCACGTAAAGGCTTTTTTG-3
shRNA3	5′-CACCGCGACATCAATCCGAACATTCTTCAAGAGAGAATGTTCGGATTGATGTCGCTTTTTTG-3′
shRNA4	5′-CACCGCTGTTTGACTTGGAGAATAATTCAAGAGATTATTCTCCAAGTCAAACAGCTTTTTTG-3′

For RNAi, cells in each well were transfected with a mixture of 0.8 μg shRNA plasmids plus 2 μl Lipofectamine™ 2000 reagents. Medium was changed with or without GBE or flavonoids 24 hours after transfection. Afterwards, cells were harvested for mRNA determination after continuous 12-hour or 24-hour incubation for western blotting analysis and cellular triglyceride content measurement.

### Electrophoretic mobility shift assay (EMSA)

Nuclear proteins were extracted using Nuclear and Cytoplasmic Protein Extraction Kit (Beyotime) after 6 hours of treatment with or without GBE. For each sample, 6 μg nuclear proteins were pre-incubated with the EMSA/Gel-Shift Binding Buffer (Beyotime) to block non-specific binding for 15 minutes at 4°C prior to the addition of the biotin-labeled probe (100 pmol) which corresponded to the −50 to −5 nt of CPT1A promoter region and further incubation for 20 minutes at 25°C. For supershifts, nuclear extracts were pre-incubated with 3 μg of antibodies against Sp1 (Abcam, Cambridge, UK) or non-immune IgG (Santa Cruz Biotechnology) (negative control) for 15 minutes at 4°C prior to addition of the labeled probe. Electrophoresis was carried out on non-denaturating polyacrylamide gels (6%) in 0.5 × TBE at 100 V for 90 minutes and then electrophoretically transferred onto a positively-charged nylon membrane in 0.5 × TBE at 380 mA for 45 minutes. The membrane was cross-linked at 120 mJ/cm^2^. The DNA-protein complex was visualized with streptavidin-HRP Conjugate (Beyotime) according to the manufacturer’s instructions.

### Chromatin immunoprecipitation (ChIP) assays

Sp1 binding activity to the CPT1A promoter region in intact cells was confirmed using a ChIP assay kit (Beyotime). Briefly, 2 × 10^7^ HepG2 cells were treated with D, G200, Q20, K20 or I8 for 6 hours and then cross-linked in 1% formaldehyde solution for 10 minutes at 37°C. Cross-linking was stopped by the addition of glycine to a final concentration of 125 mM. After 5 minutes, the cells were washed twice with 1 × PBS and harvested with PBS containing 100 mM PMSF. The cells were lysed and sonicated on ice with a Sonifier (Measuring and Scientific Equipment, UK) at 7 W for nine 10-second pulses and then centrifuged. After centrifugation, 20 μl of supernatants were used to measure total input chromatin (input control) and the rest were incubated in a rotor with 4 μg of anti-Sp1 antibody (Abcam) or normal-rabbit IgG (Santa Cruz Biotechnology) at 4°C overnight. Fifty μl of Dynabeads® Protein A beads (Invitrogen) were added for further 1.5 hours incubation the next day. Then the immunoprecipitated DNA-protein complexes were washed, eluted, and purified to conduct PCR for forty cycles. The PCR primers for ChIP assay (forward, 5′-CTCGGCGTCCCCACAG-3′; reverse, 5′-TTCCCAGGGCTCTTCG-3′) were designed to flank the Sp1 binding sites of the −50 to −5 of CPT1A promoter region, and the PCR products were analyzed on 2% agarose gel.

### Statistical analysis

Data were presented as the means ± SD. The statistical analyses were performed using an unpaired, two-tailed Student’s *t* test. The significance of differences were indicated as * *P* < 0.05 and ** *P* < 0.01.

## Results

### CPT1A enhanced the lipid-lowering effect of GBE and its flavonoids

**Figure 1:**
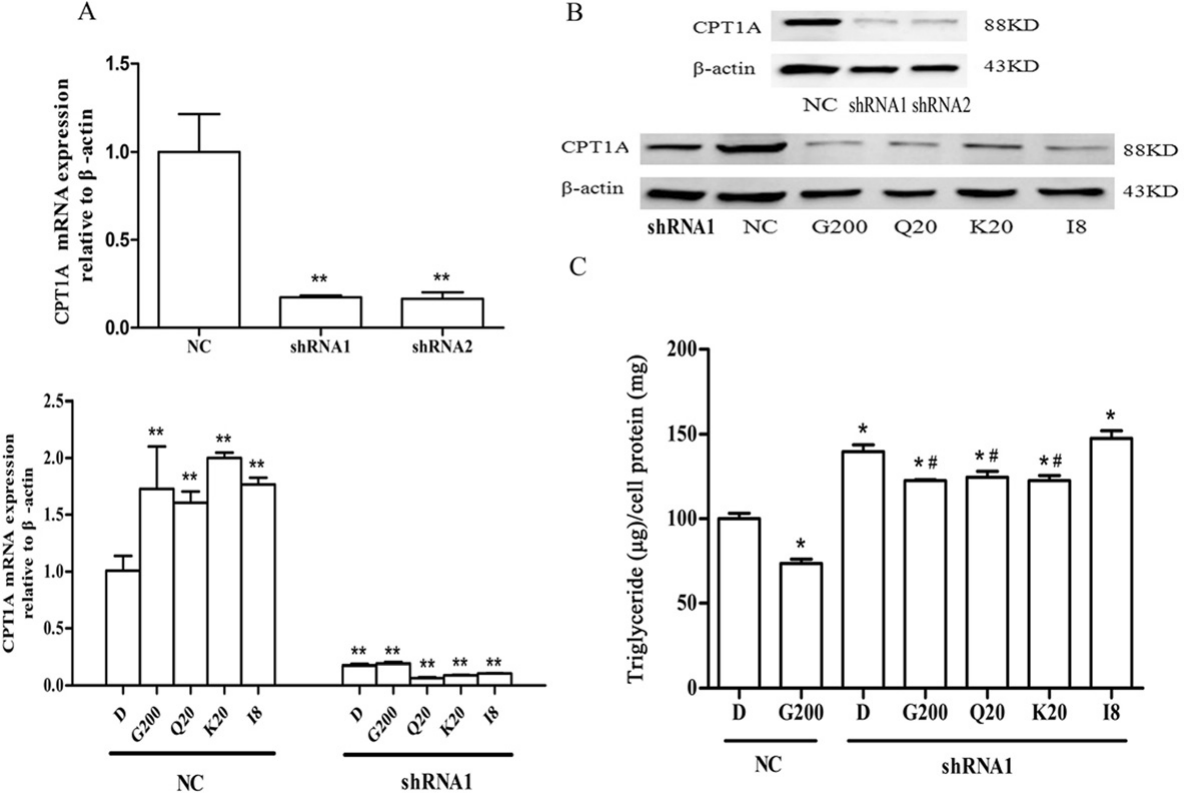
**Knockdown of CPT1A influenced the lipid-lowering effect of** ***Ginkgo biloba*** **extract (GBE) and its ingredients in HepG2 cells. (A)** CPT1A expression regulated by GBE and its flavonoids in NC- and shRNA- cells. The mRNA expression of CPT1A was normalized to β-actin. **P < 0.01 versus DMSO treatment in NC group. **(B)** CPT1A protein expression in NC- and shRNA- cells. Vectors without influence on the expression of CPT1A were negative controls (NC). **(C)** Cellular triglyceride content regulated by GBE and its flavonoids in NC- and shRNA- cells. The cellular triglyceride content in NC group with DMSO treatment was set at 100%, and values in other groups were compared to it. *P < 0.05 versus DMSO treatment in NC group. ^#^ *P* < 0.05 versus DMSO treatment in shRNA group. D, G200, Q20, K20, I8 represented HepG2 cells incubated with 0.1% DMSO, 200 μg/ml GBE, 20 μg/ml quercetin, 20 μg/ml kaempferol, and 8 μg/ml isorhamnetin, respectively.

Considering the complexity and pleiotropy of herbal medicine, we first investigated whether the lipid-lowering effect of GBE was completely dependent on CPT1A. RNA interference technology was adopted to silence endogenous CPT1A expression, and two shRNA vectors with up to 85% silence efficiency were selected out for continued experimentation (Figure 1A).

We observed a significant up-regulation of CPT1A by GBE in NC group cells when GBE or the flavonoids were added after transfection for 24 hours, while no influence was shown in the group silenced by shRNA1. The results indicated that the regulatory ability of GBE and its flavonoids on CPT1A was abolished when CPT1A was knocked down (Figure 1A). Western blotting results also confirmed a remarkable decrease in CPT1A protein and no compensation effect by drug treatment (Figure 1B). More strikingly, knockdown of CPT1A caused a high increase in cellular TG content by approximately 40% while GBE treatment slightly reduced the previous increase, suggesting the existence of alternative pathways. This regulation was, however, much weaker than that in NC cells, and cellular TG content remained notably high. In examining the three flavonoid ingredients, we found quercetin and kaempferol exhibited the similar results as GBE. In contrast, isorhamnetin lost most of the lipid-lowering effect when endogenous CPT1A’s expression was inhibited (Figure 1C). Experiments with shRNA2 achieved the same results (data not shown).

### CPT1A promoter region from −50 to −5 nt was critical for GBE-induced regulation

**Figure 2:**
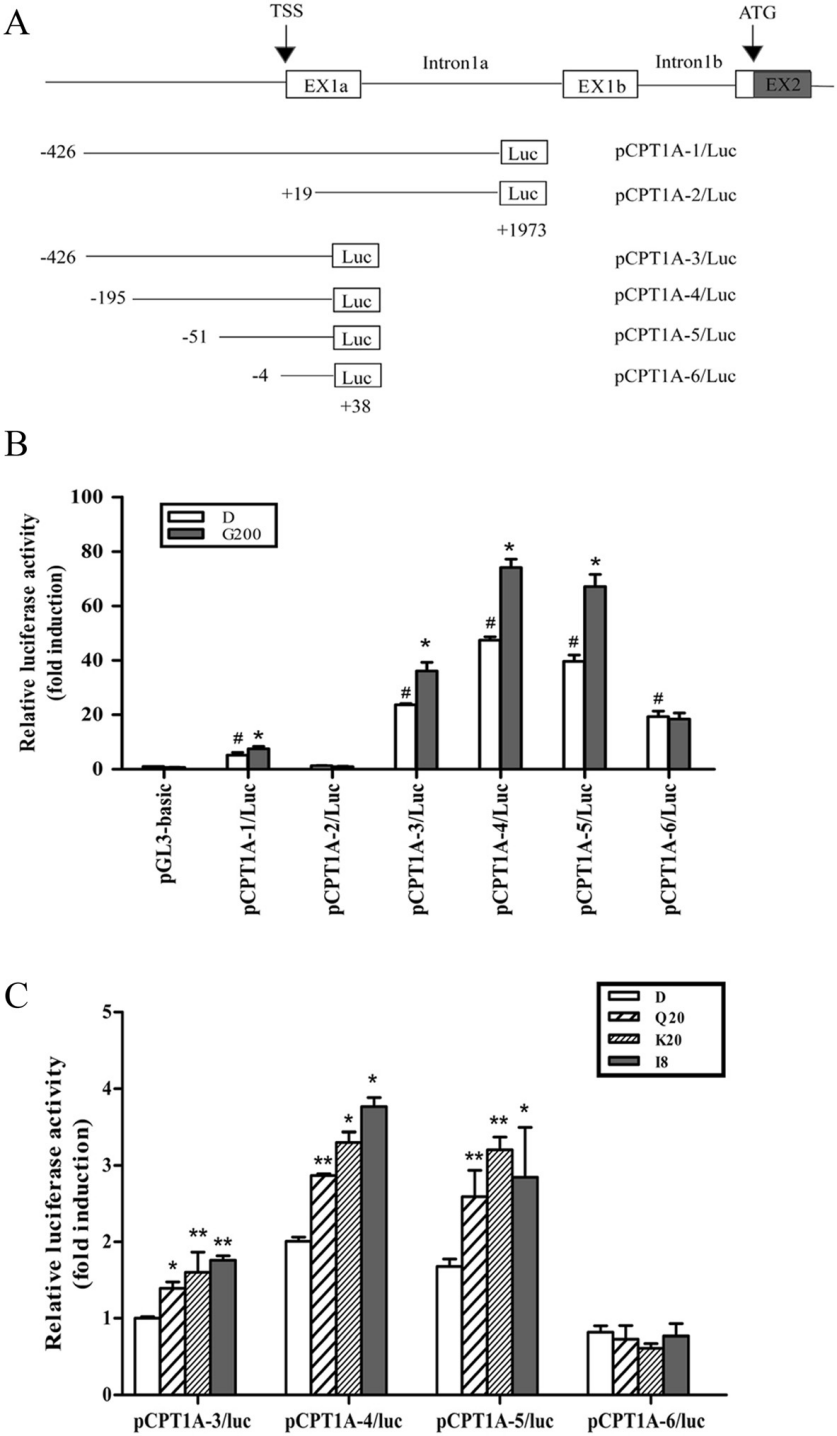
**Mapping of the GBE-responsive region in human CPT1A promoter region. (A)** A promoter fragment of the CPT1A 5′-untranslated region and a schematic representation of the constructed luciferase reporter promoters were presented. **(B)** Promoter activities of each deletion construct in the absence or presence of GBE in the HepG2 cells. Luciferase activity was presented as a fold induction relative to pGL3-basic vector whose value was set as 1. *P < 0.05 versus each respective control group (DMSO, D). ^#^ *P* < 0.05 versus DMSO treated group of pGL3-basic vector. **(C)** Regulation of each deletion promoter construct in flavonoid-treated HepG2s. The luciferase activity in each group was calculated as a fold induction relative to the untreated pCPT1A-3/Luc whose value was set as 1. *P < 0.05 and **P < 0.01 versus corresponding untreated construct.

We had confirmed previously that GBE significantly promoted the expression of CPT1A in HepG2 cells. To further define the transcriptional regulation mechanisms of CPT1A, a series of luciferase reporter plasmids were constructed (Figure 2A) and transfected into HepG2 cells. As shown in Figure 2B, in the presence of GBE, activities of pCPT1A-1/Luc and pCPT1A-3/Luc constructs were enhanced by 1.5 to 1.7 fold, indicating that the region −426 to +38 was indispensable for GBE regulation. With the pCPT1A-5/Luc (−51 to +38) construct, we observed the activity was 1.6-fold higher in the presence of GBE, while there was no such effect with the pCPT1A-6/Luc (−4 to +38) construct. Thus, we deduced that GBE-regulatory elements would be within the region −50 to −5. The same results could be obtained with the flavonoids, the active ingredients of GBE (Figure 2C).

### Mutation of Sp1 binding sites in the CPT1A promoter region affected the responsiveness of GBE

Computer-aided analysis (TESS, http://www.cbil.upenn.edu/cgi-bin/tess/tess; promoter scan, http://www-bimas.cit.nih.gov/molbio/proscan/; Tfsitescan, http://www.ifti.org/cgi-bin/ifti/Tfsitescan.pl) revealed two important putative Sp1 binding sites in the −50 to −5 of CPT1A promoter region, Sp1a (−44 to −35) and Sp1b (−18 to −9), respectively.

**Figure 3:**
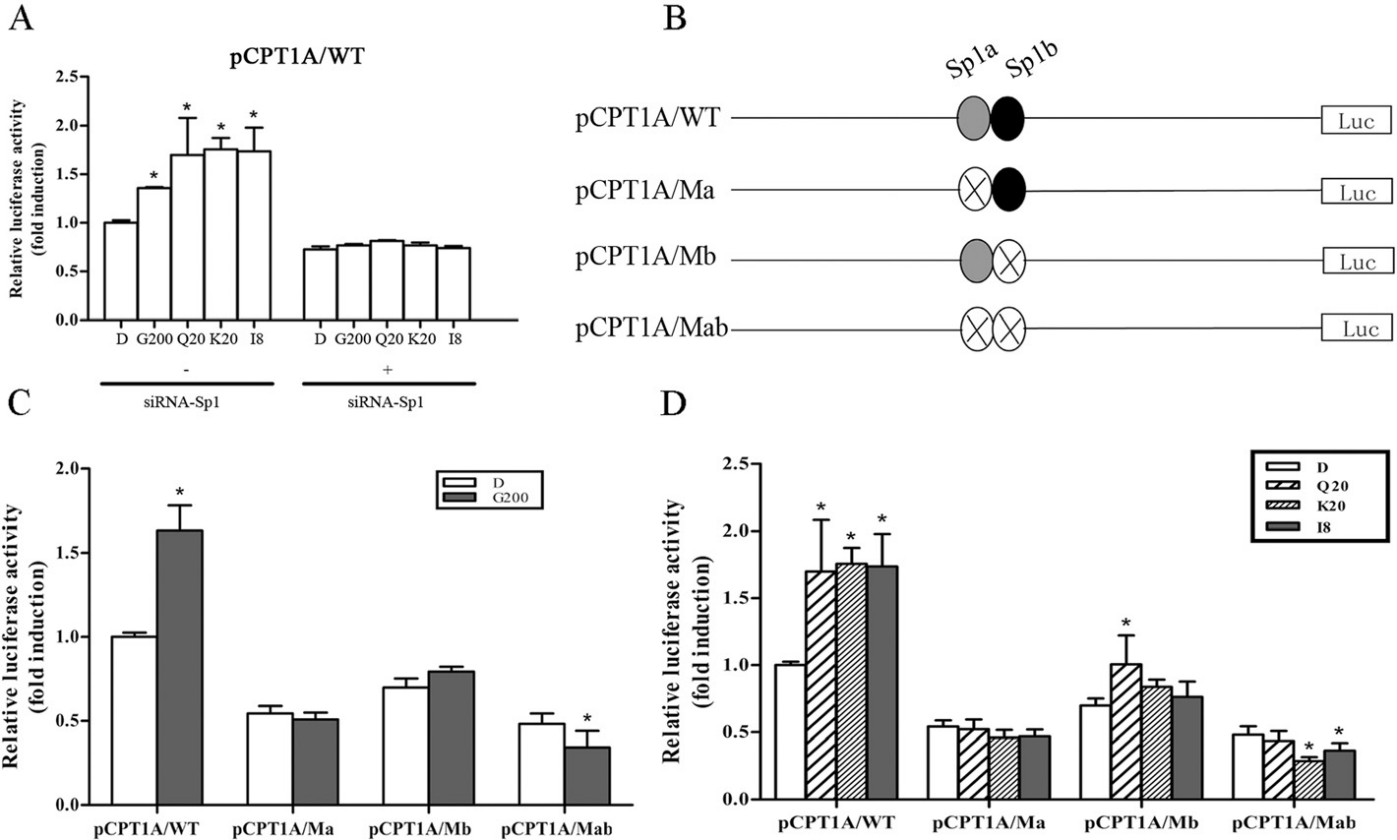
**Functional analysis of Sp1 binding sites in the promoter of CPT1A. (A)** Effect of Sp1 on activity regulation of CPT1A promoter by GBE and its flavonoids. *P < 0.05 versus respective DMSO-treated construct. D, G200, Q20, K20, I8 represent HepG2 cells incubated with 0.1% DMSO, 200 μg/ml GBE, 20 μg/ml quercetin, 20 μg/ml kaempferol, and 8 μg/ml isorhamnetin, respectively. **(B)** Illustration of constructs from region −50 to −5 with mutation of putative Sp1 sites. The filled circles represent each SP1. **(C)** Site-directed mutation analysis of Sp1 in the proximal promoter region of human CPT1A in HepG2 cells. Luciferase activity in each group was presented as a fold induction relative to untreated wild construct (value was set as 1). Each value represents mean ± SD of three individual experiments; *P < 0.05 versus each control treated group. **(D)** Mutation analysis of putative Sp1 binding sites in CPT1A promoter by flavonoids treatment. Values of luciferase activity were presented as a fold induction relative to the DMSO-treated pCPT1A/WT (value was set as 1). *P < 0.05 and **P < 0.01 versus respective DMSO-treated construct. Values were presented as mean ± SD of three experiments.

We first investigated whether GBE could induce transcription of the construct contained −50 to −5 of CPT1A promoter region (pCPT1A/WT) without Sp1. We designed an siRNA-Sp1 to knockdown Sp1 with about 60% inhibition efficiency detected by RT-PCR (Additional file 1: Figure S1A). As shown in Figure 3A, there was no improvement in activity of pCPT1A/WT when Sp1 was inhibited, while GBE highly enhanced its activity by 1.4 fold when Sp1 was present. The flavonoids -- quercetin, kaempferol, and isorhamnetin -- also showed the same results that Sp1 played an important role in this regulation.

Furthermore, to define the role of Sp1a and Sp1b in GBE-inducible CPT1A transcription, substitute mutations were introduced into their core sequences (Figure 3B). Mutation of Sp1a destroyed the responsiveness of GBE while the Sp1b mutation caused little effect. Moreover, mutation of Sp1a and Sp1b at the same time induced even more remarkable changes (Figure 3C). Investigations on the flavonoids also revealed that pCPT1A/Ma and pCPT1A/Mab constructs containing an Sp1a mutation were unresponsive to the flavonoids while the pCPT1A/Mb construct containing a mutation in Sp1b displayed little effect in induced luciferase activity (Figure 3D). Among the three ingredients, kaempferol and isorhamnetin showed a relatively higher activity than quercetin. Taken together, Sp1 sites -- especially Sp1a in the region of −50 to −5 -- were essential to the up-regulation of human CPT1A by GBE and its flavonoid ingredients.

### GBE enhanced Sp1 transfer from cytoplasm to nucleus

**Figure 4:**
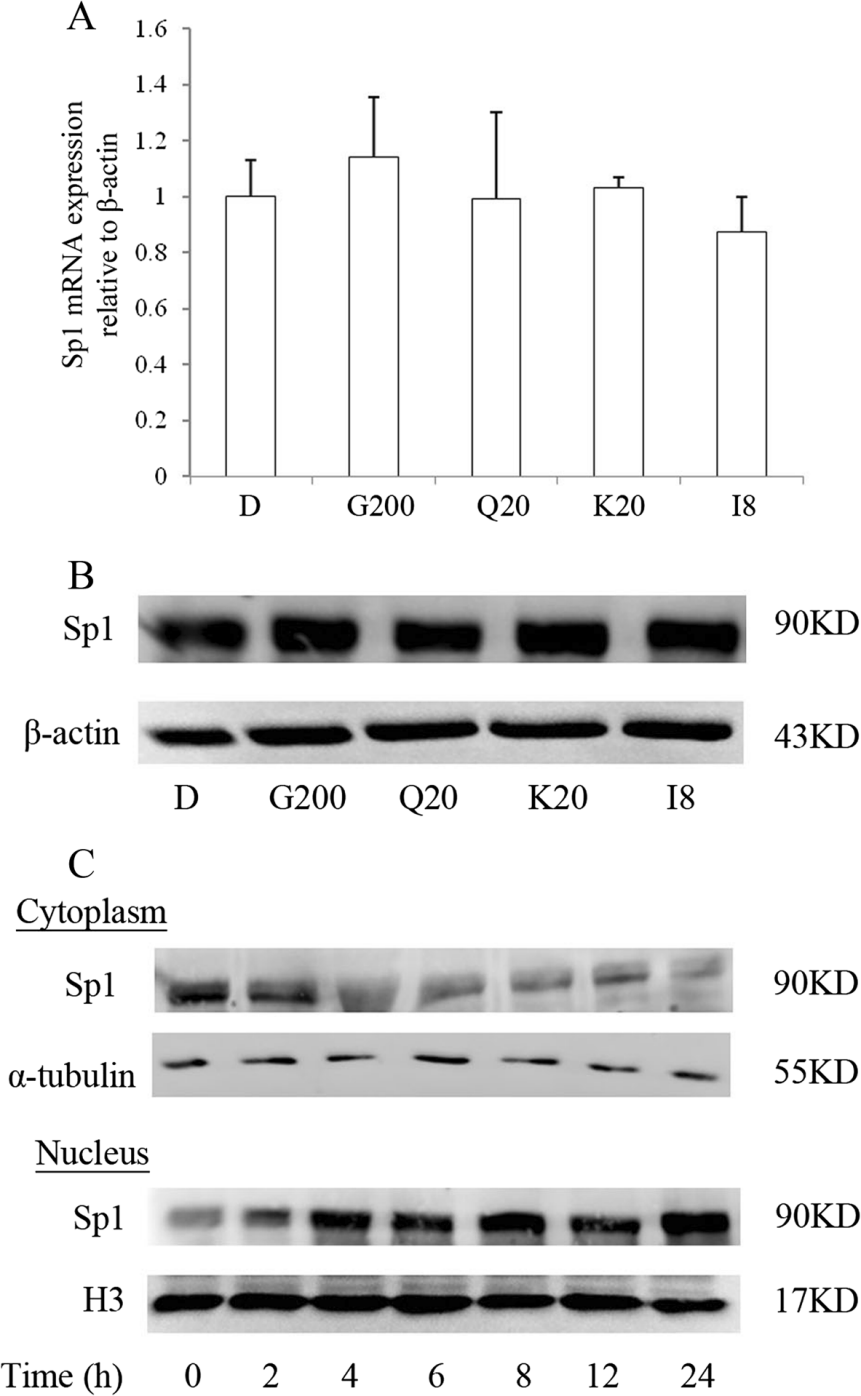
**Regulation of Sp1 by GBE. (A)** mRNA expression of Sp1 regulated by GBE in HepG2 cells. **(B)** Protein expression of Sp1 regulated by GBE in HepG2 cells. β-actin was used as control. **(C)** Expression analysis of Sp1 in cytoplasm and nucleus regulated by GBE. Expression of cytoplasmic Sp1 was normalized to α-tubulin. Expression of nucleus Sp1 was normalized to histone H3. D, G200, Q20, K20, I8 represent HepG2 cells incubated with 0.1% DMSO, 200 μg/ml GBE, 20 μg/ml quercetin, 20 μg/ml kaempferol, and 8 μg/ml isorhamnetin, respectively.

To investigate the regulation of Sp1 by GBE, RT-PCR and western blotting were performed. No striking changes were observed on whole mRNA and protein expression of Sp1 (Figure 4A and B). However, we detected an obvious transfer of Sp1 from cytoplasm to nucleus by western blotting in a time-dependent manner upon GBE treatment, especially at 24 hours (Figure 4C). Thus, GBE might increase the content of Sp1 in the nucleus through induced transfer from cytoplasm, in addition to directly enhanced its expression.

### GBE promoted Sp1 binding to the promoter region of CPT1A

**Figure 5:**
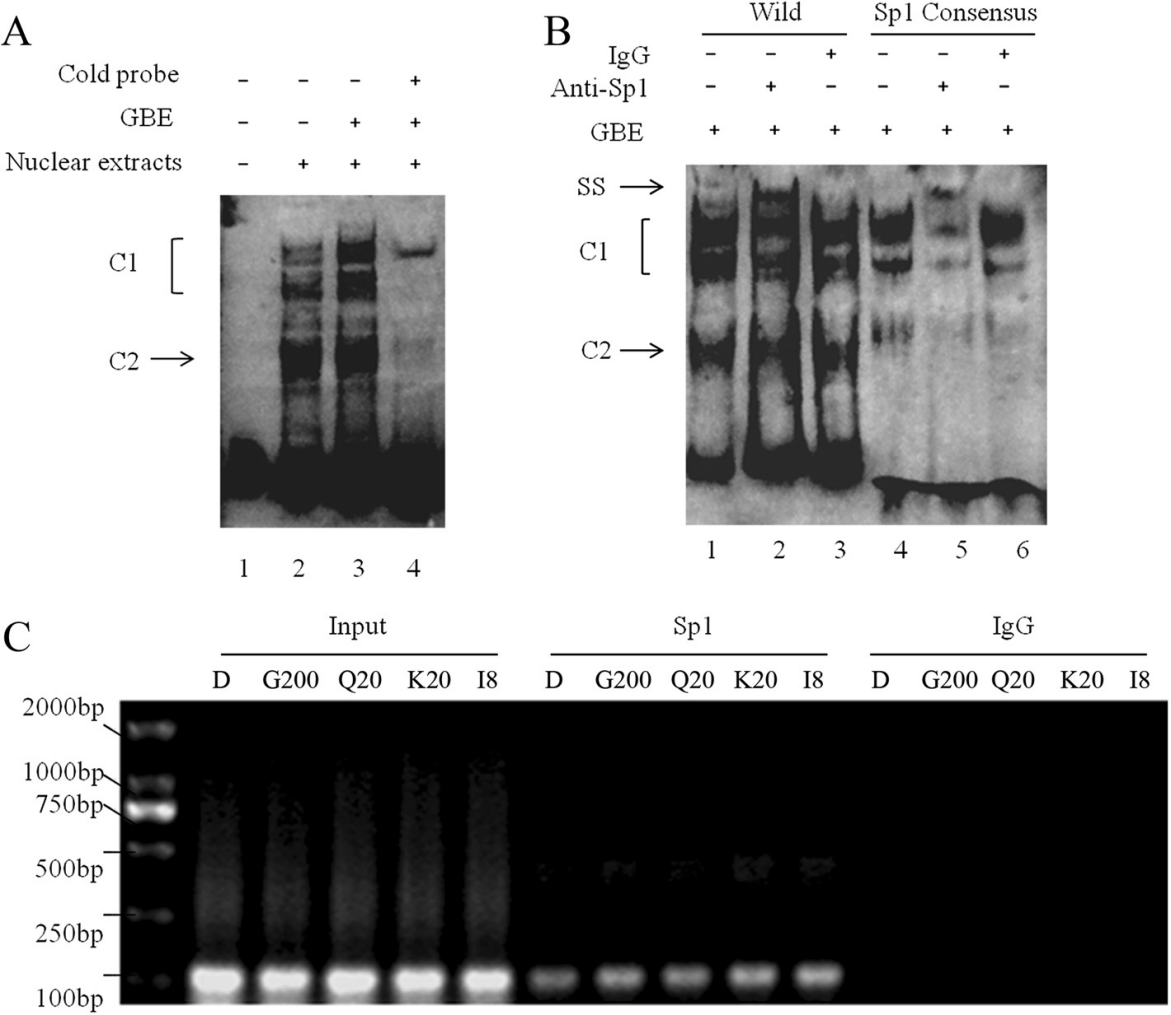
**Analysis of Sp1 response element in CPT1A promoter region. (A)** Electrophoretic mobility shift assay (EMSA) was performed for identification of Sp1 binding to the CPT1A promoter region from −50 to −5 *in vitro*. DNA-protein complexes were formed upon addition of nuclear extracts from vehicle or GBE-treated HepG2s (lane 2, 3). The binding specificity was competed by excess (×100) unlabeled (cold) probes (lane 4). **(B)** For supershift, binding reactions were carried out in the presence of antibodies against Sp1 or non-immune goat IgG (negative control). **(C)** ChIP analysis in HepG2 cells showed that GBE induced Sp1 to the indicated binding sites. Left panel, input control (for each group, 0.15 μg DNA was used for PCR); middle panel, Sp1 immunoprecipitation; and right panel, IgG immunoprecipitation (negative control). PCR was performed as described in Chromatin Immunoprecipitation Assay (ChIP) Section and products were run on a 2.0% agarose gel. D, G200, Q20, K20, I8 represent HepG2 cells incubated with 0.1% DMSO, 200 μg/ml GBE, 20 μg/ml quercetin, 20 μg/ml kaempferol, and 8 μg/ml isorhamnetin, respectively.

To determine whether or not GBE can affect Sp1 binding to CPT1A’s promoter region, EMSA was performed using nuclear extracts from HepG2 and a biotin-labeled probe covering the −50 to −5 of CPT1A promoter region. DNA-protein complex revealed bands C1 and C2 after incubation (Figure 5A, lane 2). However, GBE-treated cell nuclear lysates had significant increases in C1 formation but no obvious influence on C2 (Figure 5A, lane 3). The binding bands were specifically competed by 100-fold molar excess unlabeled (cold) probes (Figure 5A, lane 4). Above observations indicated that GBE promotes Sp1 binding to the *CPT1A* promoter region. It was also validated by supershift assays. Labeled Sp1 consensus probe was used as positive control. Anti-Sp1 antibody incubation resulted in a supershift band (ss) (Figure 5B, lane 2 and 5), while negative IgG did not (Figure 5B, lane 3 and 6). With ChIP assay, more PCR products could be detected in GBE or its ingredients treated HepG2 cells. While the input samples gave the same yields, no PCR product was detected without applying Sp1 antibody (Figure 5C).

## Discussion

Currently, although treatment of NAFLD by diet control and body weight loss results in histological improvement, pharmacotherapeutic options are lacking and remain focused on treating concurrent metabolism syndrome [14]. Our previous studies demonstrated lipid metabolism, and inflammatory and stress response-related genes were regulated through the integrated transcriptome and metabolome profiles of GBE [4,15,16].

GBE and its flavonoid components -- quercetin, kaempferol, and isorhamnetin -- are widely found in fruits and vegetables. They reduce the hepatic lipid accumulation and up-regulate the expression of CPT1A [5,17–19]. Patients with NAFLD had lower CPT1A expression and activity [20]. In addition, physiological over-expression of this rate-limiting enzyme *in vivo* and *in vitro* was sufficient to prevent the fatty acid-induced lipid accumulation and even reduce insulin resistance [11,13]. Thus, we aimed to characterize the probable molecular mechanisms by which GBE and its flavonoids regulate CPT1A expression and are hopeful in its potential benefits for treating NAFLD.

The majority of fatty acid oxidation in the liver occurs in mitochondria and is regulated by CPT1A. We silenced CPT1A in HepG2 cells through RNAi to impair the oxidative capacity of mitochondria and observed a significant increase in cellular triglyceride content. GBE and its flavonoid contents -- quercetin and kaempferol minus isorhamnetin -- lowered triglycerides without influencing CPT1A expression, which suggested there were alternative pathways at play. β-oxidation was also reported to occur in peroxisomes and microsomes [21]. GBE had also been reported to up-regulate a suite of genes related to peroxisomes and microsomes oxidation in an NAFLD rat model, such as straight-chain acyl-CoA oxidase (Acox), PPARα, and cytochrome P450 enzymes, indicators of fatty acid consumption. Quercetin and kaempferol were also confirmed to affect partial genes [5,18–20,22]. Therefore, GBE and its flavonoids might increase CPT1A expression to promote β-oxidation in mitochondria, as well as in peroxisomes and microsomes.

Human *CPT1A* is a TATA-less gene with GC-rich regions in the proximal promoter. Sp1 is a ubiquitously-expressed transcription factor belonging to a zinc finger family and has an important role in directing transcription of the TATA-less genes [23,24]. In rat, Sp1 was found to bind to the promoter of CPT1A and was responsible for driving its basal expression [25]. Our observation also confirmed Sp1 could affect the expression of CPT1A and cellular triglyceride content (Additional file 1: Figure S1B and C). In addition, we revealed for the first time a region from −426 to +38 containing cis-elements for the basal transcription of human CPT1A. Sp1 was most likely predicted to bind in region −50 to −5 through computer-aided analysis. Other factors were also predicted, for example, YY1, TBP, AP2, ERα had the ability to co-regulate with Sp1 in TATA-less genes [26–29]. Therefore, Sp1 might co-regulate basal transcription activity of CPT1A with various transcription co-factors.

We identified an indispensable segment of GBE-acting locus located at −50 to −5 with predicted Sp1 binding sites and high-GC content using deletion and mutation analysis. EMSA results demonstrated that GBE induced a marked increase in DNA-protein complex formation in this region. ChIP analysis confirmed the GBE-induced binding of Sp1 in intact cells. The flavonoid ingredients also obtained the same responsive element with GBE. These findings suggested that the presence of Sp1 binding region between −50 and −5 was important for GBE-induced regulation of CPT1A as well as the flavonoids.

Sp1 activity is significantly regulated through post-translational modifications, including glycosylation, acetylation, and phosphorylation [30,31], especially phosphorylation through ERK, JNK, and p38-MAPK signaling pathways [32,33]. Quercetin and isorhamnetin, ingredients of GBE, were found to induce genes through these pathways [34–36]. Thus, in hepatic cells, GBE and its flavonoid compounds might activate Sp1 through mitogen-activated and protein kinase-mediated signal transduction pathways.

Sp1 also proved to be involved in regulating multiple transcription processes. In human endothelial cells, it was indispensable that enhanced binding of Sp1 to the promoter region of tissue-type plasminogen activator (t-PA) in quercetin induced expression through a p38-dependent pathway [34]. Additionally, quercetin could lead to binding of Sp1 to ABCA1 [37] and significantly up-regulate DR5, a death receptor of TRAIL, in a transcription factor, Sp1-dependent manner [38]. Moreover, other flavonoids like genistein and daidzein were also reported to target genes in the GC-rich Sp1 binding sequence in intestinal cells [39]. Nobiletin, another citrus polymethoxylated flavonoid, regulated the bacterial lipopolysaccharide (LPS)-induced expression of tissue factor (TF) through interaction with Sp1 [40].

In addition to regulating Sp1, natural products could also be involved in regulating other transcription factors. Flavonoids like genistein, kaempferol, quercetin, and daidzein inhibited STAT-1 and NF-kappa B activations in anti-inflammatory effects [41]. The regulatory effect of quercetin on two main transcription factors (NF-kappa B and AP-1) was also investigated and found to related to survival/proliferation pathways in a human hepatoma cell line [42]. Other natural products like caffeic acid phenethyl ester was also reported to interact with NF-kappa B [43]. From this we could see that regulation of transcription factors might be a mechanism of action of natural products.

## Conclusion

We have shown that a direct and specific increase in CPT1A plays a crucial role in GBE and its flavonoids’ effects. GBE, as well as quercetin, kaempferol, and isorhamnetin, up-regulate CPT1A through interaction with Sp1 to promote fatty acid β-oxidation and further exert their lipid-lowering effect. Better understanding of this mechanism may provide implications in GBE application and treatment approaches for related metabolic diseases.

## Additional file

Additional file 1: Figure S1. Expression of CPT1A and the cellular triglyceride content after knockdown of Sp1 in HepG2 cells. **(A)** mRNA and protein expression of Sp1. **(B)** mRNA and protein expression of CPT1A. **(C)** The cellular triglyceride content. NC represented negative control. The cellular triglyceride content in NC group was set as 100%, and values in other groups were compared to it. **P* < 0.05 versus NC group.

## Abbreviations

GBE: *Ginkgo biloba* extract
CPT1A: Carnitine palmitoyltransferase 1A
NAFLD: Non-alcoholic fatty liver disease
TG: Triglyceride
HRP: Horseradish peroxidase
RNAi: RNA interference
EMSA: Electrophoretic mobility shift assay
ChIP: Chromosome immunoprecipitation
