# Supplementary figures and images for: Flavonoid ingredients of Ginkgo biloba leaf extract regulate lipid metabolism through Sp1-mediated carnitine palmitoyltranferase 1A up-regulation
Source: J Biomed Sci. 2014 Sep 3;21(1):87. doi: 10.1186/s12929-014-0087-x (PMC4428510; doi:10.1186/s12929-014-0087-x)

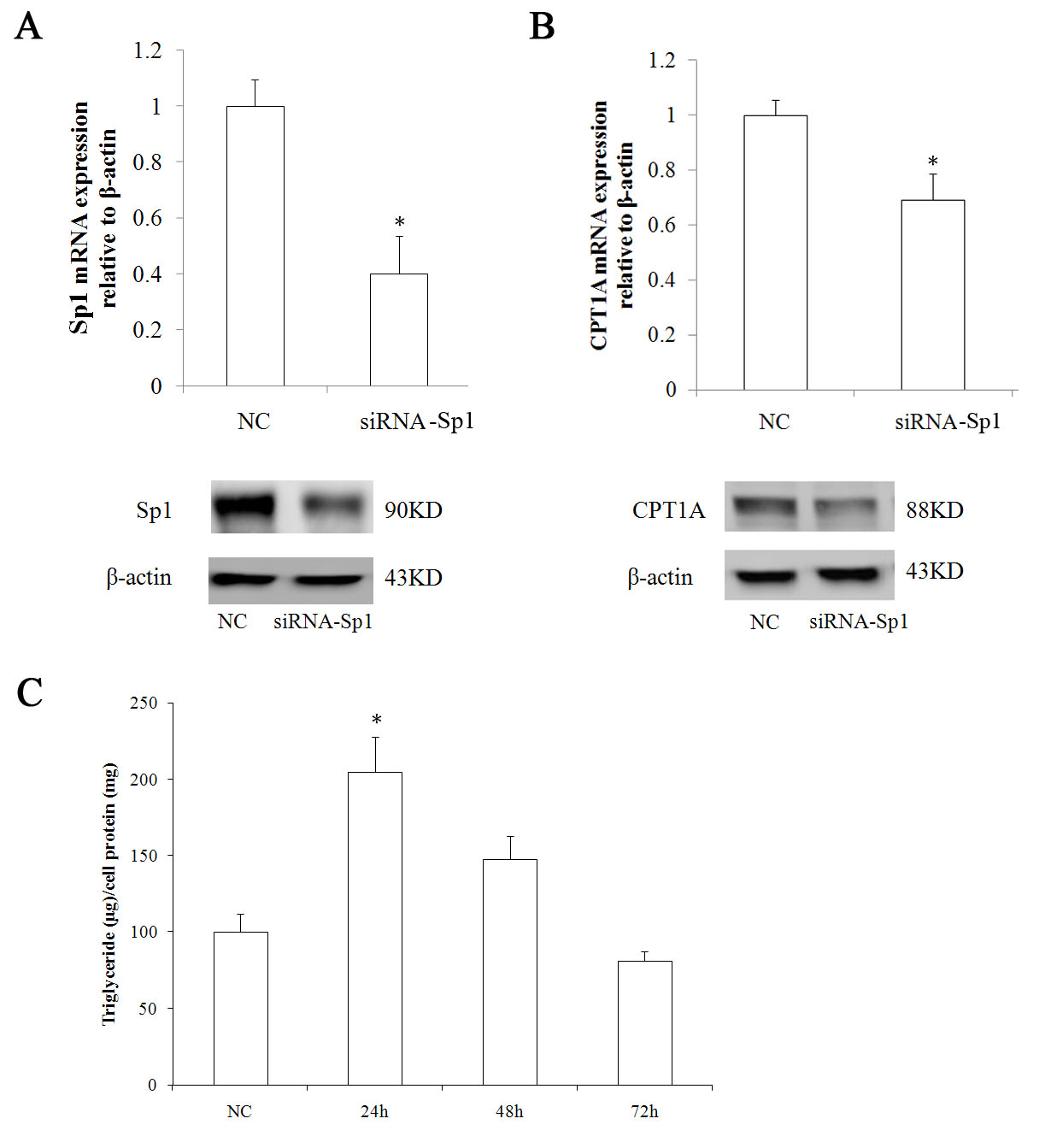

Supplement: Additional file 1: Figure S1. — Expression of CPT1A and the cellular triglyceride content after knockdown of Sp1 in HepG2 cells. (A) mRNA and protein expression of Sp1. (B) mRNA and protein expression of CPT1A. (C) The cellular triglyceride content. NC represented negative control. The cellular triglyceride content in NC group was set as 100%, and values in other groups were compared to it. *P < 0.05 versus NC group. [file 12929_2014_87_MOESM1_ESM.jpeg]
